# Sexual health knowledge and practices and STI/HIV prevalence among long-distance truck drivers in Peru

**DOI:** 10.1177/2050312117746308

**Published:** 2017-12-12

**Authors:** Patricía J García, Boris Fazio, Angela M Bayer, Aldo G Lizarraga, Marina Chiappe, Sayda La Rosa, Marcela Lazo, Lorena López, María Valderrama, César P Cárcamo

**Affiliations:** 1 School of Public Health and Administration, Universidad Peruana Cayetano Heredia, Lima, Peru; 2 Division of Infectious Diseases, David Geffen School of Medicine, University of California, Los Angeles, Los Angeles, CA, USA

**Keywords:** HIV, sexually transmitted infections, truck drivers, Peru

## Abstract

**Objectives:**

HIV and other sexually transmitted infections remain a challenge globally and many key groups have yet to be studied. Evidence shows that truck drivers may have high-risk behaviors and higher sexually transmitted infection/HIV prevalence because they are a highly mobile population. However, there is little to no information on this group in Peru. Therefore, we explored the sexual health knowledge and practices and carried out sexually transmitted infection/HIV testing among male truck drivers and their assistants in Peru.

**Methods:**

We conducted a cross-sectional study utilizing cell phone-based behavioral surveys and sexually transmitted infection testing, including HIV, syphilis, gonorrhea, and chlamydia, with truck drivers and their assistants who were traveling on two major international highways in Peru.

**Results:**

A total of 1150 truck drivers and assistants participated. Participants were middle-aged men (average age = 39.8 years), 96.0% had complete secondary education, 78.4% were in stable relationships, and 88.7% earned more than minimum wage. The majority were aware of sexually transmitted infections/HIV, but very few recognized sexually transmitted infection symptoms. Few participants (under 5%) reported recent sexually transmitted infection symptoms. Prevalence of sexually transmitted infections was also low: no one had gonorrhea; 0.1% had HIV; 0.4% had recent syphilis infection (rapid plasma reagin ≥1:8); and 2.0% had chlamydia. The prevalence of these diseases is not different from that of the general population in Peru.

**Conclusion:**

When compared to other truck drivers worldwide, Peruvian truck drivers appear to have a lower risk of HIV/sexually transmitted infections. This may be since Peruvian drivers are older, more educated, have higher income, and spend fewer days away from home than their peers globally.

## Introduction

Sexually transmitted infections (STIs) and HIV are important global health problems.^1–6^ There are certain groups that are more vulnerable to STIs/HIV, including long-distance truck drivers. Globally, there is a wide recognition of the association between migration and mobility and STIs/HIV.^7^ Long-distance truck drivers need to travel frequently and for extended periods of time, often leaving behind a partner. When surveyed, truck drivers in Africa,^8,9^ Brazil,^10,11^ and India^12^ reported sexual encounters during their trips. Truck drivers could then transmit the STI/HIV they may acquire during these encounters to their stable partners^13^ or even alter the prevalence of these diseases in the communities along their routes.^14^ Additionally, HIV prevalence was found to be higher among long-distance truck drivers than the general population, for example, in India (2.6% vs 0.3%).^15^ Finally, studies have shown that truck drivers have limited exposure to STI/HIV prevention programs.^16–18^

There have been very few studies in Latin America about long-distance truck drivers and STI/HIV risk.^17^ There have been several studies with truck drivers in Brazil.^10,11,19^ The only study to date in Peru is a Master’s thesis by Peceros (unpublished, 2006) with long-distance truck drivers at the Jahuay checkpoint (also included in this study). This study found that drivers had limited knowledge about STIs and reported frequent casual and commercial sex, with inconsistent condom use. The study did not measure STI/HIV prevalence. In a recent qualitative study with women living with HIV conducted by our research group, many women identified and related their HIV status to their partners’ job and specifically to their partners working in transportation including long-distance trucking.^20^

In Peru, there is no information on STI/HIV prevalence among long-distance truck drivers and limited information on STI-/HIV-related knowledge, attitudes, and practices among truck drivers. Furthermore, there are no prevention interventions targeting this population. However, there has been an increase in new roads and highways in the country.^21^ Therefore, we conducted a survey about sexual health knowledge and practices and carried out STI/HIV testing among male truck drivers and their assistants traveling on two of the country’s largest highways.

## Methods

We carried out this cross-sectional study in collaboration with the National STI/HIV Program of the Peruvian Ministry of Health (MOH) and authorities from the private and public transportation sectors. Fieldwork was conducted in August and September 2014.

### Population and context

There were two data collection sites where truckers stop to eat or rest: the Jahuay toll booth in southwestern Peru on the coast, located at 190 km of the South Pan-American highway in the Ica region, and the Puerto Maldonado trucker pit stop in southeastern Peru in the jungle, located at 430 km of the South Interoceanic highway in the Madre de Dios region. These are two of the three major international highways in Peru.^22^

This study included truck drivers and their assistants according to the following inclusion criteria. Truck drivers had to be aged 18 years or older, be driving a truck with three or more axels, have a professional driver’s license (A3 class), and agree to voluntarily participate. Assistants had to be aged 18 years or older and could participate only if the driver participated as well. Only males were selected, as it is very unusual for females to be truck drivers or assistants. For each site, a sample of 800 participants (expecting 400 drivers and 400 assistants) would produce a maximum error of 2.4% for stratum-specific STI prevalence no larger than 6%. Thus, the target sample size was 1600.

### Data collection methods and procedures

We approached the truckers at the two study sites. A census enumerator registered all trucks parked at the locality by writing down the license plate numbers of trucks with three or more axels. The team visited the trucks door by door and also looked for truck drivers at nearby gas stations or restaurants to invite them and their assistants to participate. After explaining the study, if the person was interested, the research team carried out the informed consent process, allowing time for questions and securing written consent.

The study team enrolled a consecutive sample of drivers and their assistants daily during 20 h of each 24-h period. We used face-to-face interviews to administer questionnaires programmed into cell phones using Magpi (Washington, DC). Survey questions on *sexual health knowledge* focused on knowledge of STIs, including general awareness and syptoms, and awareness of where to obtain condoms. Survey questions on sexual behavior asked about lifetime and recent sexual behaviors, including alcohol and drug use during sex and condom use. Questions were based on previous questionnaires used by our study team in Peru, including the PREVEN study.^4^ The survey also explored sociodemographic characteristics and travel for work.

### STI/HIV testing

We also offered STI/HIV testing. First, participants received pre- and post-test counseling in accordance with national guidelines.^23^ Then, we requested a finger-prick blood sample for a rapid dual HIV/syphilis test (Bioline^®^; Standard Diagnostics, Korea), for which results were available in 20 min. We requested a venous blood sample from participants with reactive HIV rapid test results for confirmatory testing (enzyme-linked immunosorbent assay (ELISA) and western blot) and from those with positive syphilis rapid test results for a second rapid test performed at the laboratory and for rapid plasma reagin (RPR) testing. Those with positive syphilis rapid test results were asked about previous syphilis diagnoses and treatment and offered 2 g of azithromycin to be taken orally.^24^ When the results of the rapid syphilis test performed in the field were positive and the test in the laboratory was negative, the Treponema pallidum hemagglutination assay (TPHA) test was performed. The diagnosis of recent syphilis was assigned to samples with confirmed positive tests for syphilis and a titer ≥1:8, in accordance with other studies in Peru and internationally. Finally, we requested urine samples for gonorrhea and chlamydia testing. Urine samples were stored in a cooler place, transported to a local laboratory where they were aliquoted and frozen to −20°C, and sent to the STI Laboratory of the Universidad Peruana Cayetano Heredia (UPCH). Samples were screened with the Aptima Combo 2 test (Hologic Inc., Marlborough, MA). This same laboratory also carried out the confirmatory HIV testing and RPR tests.

All drivers and their assistants received a brochure about STI/HIV prevention. They were given the option to receive their test results via text message or pick them up at their preferred MOH health establishment. Results were available within 2 weeks. Text messages with test results were general and did not refer specifically to STIs. Those with positive results also received phone calls.

### Analysis

To assess knowledge, we determined whether participants had heard about HIV, AIDS, and STIs; knew how to prevent HIV/STIs; could recognize STI symptoms; and could identify where to get condoms. To assess practices, we determined age at sexual debut, number of sex partners (lifetime and last 3 and 12 months), gender and type of relationship with sex partners, type of sexual intercourse, alcohol and drug use during sex, and condom use.

Questionnaires were applied using cell phones, which eliminated transcription errors and permitted georeferencing and real-time data consistency verification and quality control. STATA version 8 (College Station, TX) was used to calculate frequencies, percentages, and distributions of variables. For STI/HIV prevalence, we present 95% confidence intervals with Poisson distribution.

### Ethics

The ethics committee at the UPCH reviewed and approved the study protocols and instruments.

## Results

During the study period, there were 3601 sightings of eligible trucks. After reviewing the results of the truck census, it was determined that 2500 different trucks were registered.

A total of 1390 truckers and assistants were contacted. Of these, 8 did not want to provide their eligibility data and 4 were ineligible (2 were assistants of drivers who did not participate, 1 did not have an A3 license, and 1 was underage). Of the remaining 1378 eligible candidates, 213 did not want to participate for the following reasons, with more than one reason allowed per person: 163 due to lack of time, 26 since they reported that their own health care provider provides their health check-ups, 13 due to fear of needles, 3 due to fear of a confidentiality breach, and 23 for other reasons. After the consent process, 1165 agreed to participate (84.5% of eligible drivers/assistants identified). We have data for 1150 who responded to the questionnaire, 1113 who underwent rapid STI testing, and 1083 who gave urine samples. There were 13 participants who provided biological samples but chose not to provide questionnaire data. There were 2 consenting participants who withdrew their consent afterwards and decided not to provide questionnaire data or biological samples.

### Participants’ general characteristics

The average age was 39.8 years (Table 1). Secondary education is a requirement for obtaining a driver’s license in Peru, which resulted in 96.0% of drivers having at least this level of education. Most participants were married or in common law marriages (78.4%). The great majority (97.4%) were Peruvian and of the non-Peruvians, 25 were Bolivian, 3 Brazilian, 1 Chilean, and 1 Salvadoran. Finally, we included mainly drivers (89.4%) and a small group of assistants.

**Table 1.** Sociodemographic and work-related characteristics of participants, long-distance truck drivers and assistants, n = 1150, Jahuay/Ica and Puerto Maldonado, Peru, 2014.

	Total, n (%)
Age (years), mean (range)	39.8 (18–72)
Level of education	
Primary or less	46 (4.0)
Secondary	851 (74.1)
Post-secondary	252 (21.9)
Marital status	
Married/common law	902 (78.4)
Widow/divorced/separated	66 (5.7)
Single	182 (15.8)
Years as truck driver, mean (range)	14.1(0–54)
Ownership of truck (only drivers)	
Owner	140 (13.6)
Company	843 (81.8)
Monthly income in Peruvian soles (US dollars)^a^	
<1000 (<359)	129 (11.3)
1000 to 1999 (359 to 718)	575 (50.6)
2000 to 3000 (718 to 1077)	367 (32.3)
>3000 (>1077)	66 (5.8)

a Exchange rate of S/2.845 per US$1 on 29 August 2014.

Minimum vital wage in 2014: US$263.62.

### Participants’ professional characteristics

The average length of time as a truck driver was 14.1 years, with a wide range of 0–54 years. Among drivers, the majority (81.8%) drove a company-owned truck. Almost all participants (88.7%) earned more than 1000 Peruvian Soles (US$359) per month, higher than the vital minimum wage at the time of the study, 750 nuevos soles or US$269 (Table 1).

Most participants (98.3%) reported interprovincial routes and a minority (21.4%) reported international routes in the past year. The current trip kept or would keep drivers away from home for a median of 6 days, with a large range of 0 days to 1 year. The median number of nights at home during an average month was 7 nights, with a range of 0 to 30 nights (data not in tables).

### Knowledge and practices related to HIV and STIs

The majority had heard about STIs (93.4%), HIV (93.6%), and AIDS (96.5%) (Table 2). However, we observed important limitations regarding recognition of STI symptoms. The most recognized symptom, burning sensation while urinating, was named by only 33.0% of participants. Urethral discharge was recognized by 17.7%, itchy external genitals or pubic lice by 11.5%, and the remaining symptoms by less than 10% of participants.

**Table 2.** STI/HIV knowledge of participants, long-distance truck drivers and assistants, n = 1150, Jahuay/Ica and Puerto Maldonado, Peru, 2014.

	Total (n (%))
Has heard about STIs	1074 (93.4)
Recognizes STI symptoms	
Urethral discharge	204 (17.7)
Burning sensation while urinating	380 (33)
Genital ulcers	101 (8.8)
Inguinal swelling	35 (3.0)
Genital warts	37 (3.2)
Itchy external genitals/pubic lice	132 (11.5)
Reports valid forms of preventing STIs	
Condom use	1013 (88.1)
Abstinence	141 (12.3)
Monogamy	231 (20.1)
Has heard of HIV	1076 (93.6)
Has heard of AIDS	1110 (96.5)
Knows HIV is preventable	1086 (94.4)
Reports valid forms of preventing HIV	
Condom use	998 (86.7)
Abstinence	115 (10.0)
Monogamy	281 (24.4)

STI: sexually transmitted infections.

Condoms were widely recognized as an STI/HIV prevention method, although about 1 in 10 drivers did not name condoms as a form of prevention (Table 2). The same proportion named abstinence and about 2 in 10 respondents named monogamy as a means of preventing HIV/STI. Pharmacies were the most frequently cited source of condoms (85.1%), followed by health establishments (9.4%) and gas stations (5.6%) in a distant second and third. Other potential sources for condoms such as supermarkets, smaller grocery stores, sex workers, hotels, friends, and bars were named by under 5% of truck drivers (data not shown).

Drivers started their sex lives at an average age of 16.2 years and had a median of 6 lifetime sexual partners with a wide range of 0 to 500 partners (Table 3). They reported a median of 2 sexual partners in the last 12 months (range: 0–80) and 1 female partner in the last 3 months (range: 0–12). Very few respondents (0.3%) reported recent sex with another man and a much higher percentage (15.3%) reported sex with a female sex worker in the last 3 months. Reported consistent condom use was lowest (8.2%, 89/1089) with regular partners, intermediate (57.1%, 450/788) with occasional partners, and highest (89.7%, 442/493) with sex workers. For the great majority (77.9%), their most recent sex was with their stable partner; although for 15.8%, it was with a casual partner (data not shown). The last sexual contact was almost always vaginal, and a small proportion reported sex under the influence of alcohol or drugs. Condom use during the last sexual encounter varied depending on the partner relationship, ranging from 15.0% with stable partners to 94.2% with a sex worker.

**Table 3.** Lifetime and recent sexual practices of participants, long-distance truck drivers and assistants, n = 1150, Jahuay/Ica and Puerto Maldonado, Peru, 2014.

	Total, n (%)
Age at sexual initiation, mean (standard deviation)	16.2 (2.4)
Lifetime number of sexual partners, median (range)	6 (0–500)
Number of sexual partners in the past 12 months, median (range)	2 (0–80)
Last 3 months	
Number of sexual partners, median (range)	1 (0–12)
Sex with female sex worker	176 (15.3)
Sex with another man	4 (0.3)
Last sexual encounter	
Stable partner	897 (78.6)
Condom Used	15.0%
Casual partner	182 (16.0)
Condom Used	45.9%
Paid partner	50 (4.4)
Condom Used	94.2%
Other type of partner	12 (1.0)
Vaginal sex	1139 (99.0)
Alcohol use	122 (10.6)
Drug use	6 (0.5)

### STI symptoms and prevalence

Very few participants reported STI symptoms at the time of the study or in the last year. During the last 12 months, urethral discharge (4.6%) and genital ulcers (4.5%) were more common than genital warts (2.0%; data not shown).

In all, 26 participants (2.3%) had a positive result on the rapid syphilis test, and after RPR confirmation, the prevalence of recent syphilis (RPR ≥ 1:8) was 0.4% (95% confidence interval (CI): 0.1–0.9; Table 4). Of the 3 participants (0.3%) with a reactive result on the rapid HIV test, only 1 was confirmed through ELISA and Western Blot (prevalence 0.1%, 95% CI: 0.0–0.5). There were no cases of gonorrhea and 22 participants (2.0%, 95% CI: 1.3–3.1) had chlamydia.

**Table 4.** Prevalence of sexually transmitted infections (STIs), long-distance truck drivers and assistants, n = 1113, Jahuay/Ica and Puerto Maldonado, Peru, 2014.

	Total, n (%) (95% confidence interval)
n = 1113	
Syphilis (rapid test)	26 (2.3)
Recent syphilis (RPR ≥ 1:8)^a^	4 (0.4)
	(0.1–0.9)
HIV (rapid test)	3 (0.3)
HIV (ELISA and western blot)	1 (0.1)
	(0.0–0.5)
n = 1083	
Gonorrhea (TMA)	0 (0.0)
	(0.0–0.3)b
Chlamydia (TMA)	22 (2.0)
	(1.3–3.1)

TMA: transcription-mediated amplification; ELISA: enzyme-linked immunosorbent assay; RPR: rapid plasma reagin.

a Corrected for three people with positive rapid test who did not provide a venous blood sample.

b One-sided 97.5% confidence interval.

## Discussion

Truck drivers here were on average middle aged, with almost universal complete secondary school, and over two-thirds report a wage higher than the minimum vital wage in Peru. They spent an average of 23–24 nights per month on the highway. The vast majority knew about STIs/HIV, but very few could name STI symptoms. Most referred to the condom as the main STI/HIV prevention method and pharmacies as their main source for condoms. Even though most had few recent sexual partners, 15% reported paying for sex in the last few months. STI/HIV prevalence in this study was lower or equal to that of the general Peruvian population.

The characteristics of the truck drivers in our study differ from those in studies of truck drivers in other contexts. In these studies, truck drivers’ level of education was much lower (only about 50% had completed high school) and their income averaged less than the vital minimum wage in their respective countries,^8,9^ and they were also younger or had more varied ages.^8,12,25^ All of these factors in our study, higher education, wages, and ages, could help explain why Peruvian truck drivers’ STI/HIV prevalence are not different from those of the general population. In contrast and as an example, a study among truck drivers in India reported a relationship between lower age and higher exposure to high-risk behaviors, as well as higher STI/HIV prevalence in the younger subgroup.^26^

Relationship status and time spent away from home among truck drivers in Peru is similar to that of truck drivers in other countries. Truck drivers in our study reported primarily stable partnerships, similar to results among truck drivers in Asia and Africa.^8,26^ The median number of nights on the road on their present route and away from home in an average month in this study are also similar to those found in studies with long-distance truck drivers in other countries.^25,27^ In a study in South Africa, there was an association between longer time on the road and higher STI/HIV prevalence. Lengthy amounts of time on the road was the main reason South African drivers reported for seeking occasional sex with sex workers or casual partners.^9^ Further research is needed into why our participants, despite similar relationship characteristics and time away from home, avoided potential high-risk practices and the associated consequences.

The STI/HIV prevalence of participating drivers was not different from that of the general population in Peru.^4^ This finding coincides with studies in Brazil^11,28^ and differs from those in Africa and Asia, where HIV/STI prevalence was much higher among long-distance truck drivers than the general population.^25,26^ It is important to note that most studies in which STI/HIV prevalence was higher among truck drivers than the general population were conducted in areas with generalized HIV epidemics (Sub-Saharan Africa and Northeastern China). In Peru, the HIV epidemic is concentrated among men who have sex with men (MSM) and transgender women.^3^ Notably, very few participants in our study reported sex with another man.

We believe that it is important to study possible differences between truck drivers with assistants and those who travel alone. Long periods of time alone have been considered by truck drivers in other studies to be a factor that pushes drivers to seek occasional sexual encounters.^9^ In addition, there are studies that show that reducing “alone time” in turn reduces high-risk behaviors.^8^

We have explored a population in our country that has not received attention as a potential key group for STIs/HIV. During this study, most truck drivers were very enthusiastic about participating and learning about how to take care of their health. They also requested an intervention with them and others who work in their sector as there is no adequate health system for them. A model to follow is that of China, which adopted a system for STI/HIV control and surveillance for long-distance truck drivers. An evaluation found that STI prevalence was lower among truckers in zones with the system.^25^ If such a system is created in Peru, the Ministries of Health and Transport, local municipalities, the truck drivers’ associations, the companies holding the highway concessions and other private sector establishments (truck companies, gas stations, and restaurants) should work together to organize a system for STI/HIV surveillance and services and other health issues that truck drivers face. Services should include point-of-care tests (like the dual syphilis and HIV test) and flexible provision of results using technology (e.g. cell phones, interconnected electronic medical records). It would also be important to explore the possibility of developing business models to offer these services.
